# Percutaneous nephrostomy under ultrasound guidance

**DOI:** 10.4103/0971-4065.78086

**Published:** 2011

**Authors:** R. K. Baishya, D. R. Dhawan, J. Jagtap, R. Sabnis, M. R. Desai

**Affiliations:** Department of Urology, Muljibhai Patel Urological Hospital, Nadiad, Gujarat, India

Sir,

We read the article “Percutaneous nephrostomy by direct puncture technique: An observational study” by Karim *et al*. with great interest.[1] Percutaneous renal access can be achieved under fluoroscopic control or using an ultrasonography (US)-guided puncture. At our institution, we also do percutaneous nephrostomy under ultrasound guidance. The shortcomings and side effects of extensive radiation during therapeutic procedures are well known. The choice of method for the type of access depends on training and personal preference. The advantages of US-guided puncture are avoidance of radiation, avoiding adjacent and visceral injury and, most importantly, intrarenal vascular injury. US offers the shortest and straight access to the collecting system with minimal morbidity. We believe that the US-guided puncture has a significant reduction in complications. The available ultrasound probes come with a puncture attachment and, on US scanning, the puncture pathway is represented by an electronic dotted line on the scanner screen, which facilitates the exact placement of the needle. US-guided access is optimal with a needle guide, because the electronic dotted line helps in assessing the depth and plane of the puncture needle. This helps in reaching the desired calix in the most accurate way.[2] Also in certain situations like patients with cardiorespiratory compromise, doing a percutaneous nephrostomy in the lateral decubitus or supine position might be a good option as minimizes the hemodynamic and respiratory risks.[3,4] Although a pigtail catheter gives better patient tolerability, it is not ideal in situations where blood, mucus, pus, or stone is expected to pass because of its small caliber. Malecot tubes provide large-bore drainage after percutaneous renal surgery, and are useful if repeat nephroscopy is planned.[5,6] We also feel that the choice of the puncture of calyx will depend on the position of the stone as the same tract can be utilized to remove the stone on a later date.
